# Improved Bending Strength and Thermal Conductivity of Diamond/Al Composites with Ti Coating Fabricated by Liquid–Solid Separation Method

**DOI:** 10.3390/ma17071485

**Published:** 2024-03-25

**Authors:** Hongyu Zhou, Qijin Jia, Jing Sun, Yaqiang Li, Yinsheng He, Wensi Bi, Wenyue Zheng

**Affiliations:** 1 National Center for Materials Service Safety, University of Science and Technology Beijing, Beijing 100083, China; heyinsheng@ustb.edu.cn; 2 Beijing System Design Institute of Electro-Mechanic Engineering, Beijing 100039, China; jqj627@sina.com; 3 Beijing Hangxing Machinery Co., Ltd., Beijing 100013, China; winlxc2@sina.com; 4 Institute for Advanced Materials and Technology, University of Science and Technology Beijing, Beijing 100083, China; yqli0677@163.com; 5 National Academy of Forestry and Grassland Administration, Beijing 102600, China; biwensi2008@163.com

**Keywords:** diamond/Al composite, liquid–solid separation (LSS), Ti coating, interfacial bonding, bending strength, thermal conductivity

## Abstract

In response to the rapid development of high-performance electronic devices, diamond/Al composites with high thermal conductivity (TC) have been considered as the latest generation of thermal management materials. This study involved the fabrication of diamond/Al composites reinforced with Ti-coated diamond particles using a liquid–solid separation (LSS) method. The interfacial characteristics of composites both without and with Ti coatings were evaluated using SEM, XRD, and EMPA. The results show that the LSS technology can fabricate diamond/Al composites without Al_4_C_3_, hence guaranteeing excellent mechanical and thermophysical properties. The higher TC of the diamond/Al composite with a Ti coating was attributed to the favorable metallurgical bonding interface compounds. Due to the non-wettability between diamond and Al, the TC of uncoated diamond particle-reinforced composites was only 149 W/m·K. The TC of Ti-coated composites increased by 85.9% to 277 W/m·K. A simultaneous comparison and analysis were performed on the features of composites reinforced by Ti and Cr coatings. The results suggest that the application of the Ti coating increases the bending strength of the composite, while the Cr coating enhances the TC of the composite. We calculate the theoretical TC of the diamond/Al composite by using the differential effective medium (DEM) and Maxwell prediction model and analyze the effect of Ti coating on the TC of the composite.

## 1. Introduction

The advent of advanced electronic devices such as insulated gate bipolar transistors (IGBTs), phased array radars, and high-power solid-state lasers has escalated the heat flux per unit area generated by chip computing, necessitating efficient heat dissipation for stable operation [1,2,3]. Higher TC than existing thermal management materials (such as Invar, Cu/W, Si/Al, SiC/Al, etc.) is needed to meet the urgent heat dissipation requirements of large-scale integrated circuits [4,5]. Although the TC of pure Al can reach 237 W/m·K, this is sufficient for conventional electronic packaging environments. But the coefficient of thermal expansion (CTE) of Al is 23.0 × 10^−6^/K, which is too large to match that of the chip (i.e., 4.0~7.0 × 10^−6^/K) [6]. This mismatch causes stress between the packaging material and the chip when the temperature changes, causing damage to the computing components [7]. The diamond with the CTE of 1.0~3.0 × 10^−6^/K [8] composited to the Al matrix to perfectly match the CTE of the chip. Diamond/Al composites emerge as promising candidates for advanced thermal management due to their matching CTE, superior TC, and low density [9,10].

However, the significant disparity in the physical and chemical characteristics between diamond particles and the Al matrix poses a challenge, leading to interface incompatibility that significantly reduces the TC of diamond/Al composite [11,12]. Furthermore, in conventional preparation methods (such as powder metallurgy [13], gas pressure infiltration [9], vacuum pressure infiltration [14], vacuum hot pressing [15], and spark plasma sintering [16]), the interface of the two phases inevitably produces Al_4_C_3_ under high-temperature or long-term contact conditions [9,13,14,15,16]. The presence of Al_4_C_3_ at the interface not only significantly decreases the TC of the composite [17] but also restricts its application due to the hydrolytic nature of Al_4_C_3_ [9,18]. Previously, it was believed that the diamond surface coating could enhance the interface bonding between the diamond and Al, improve TC, and avoid the formation of Al_4_C_3_. However, it has recently been found that even with an intact diamond coating, minor variations in preparation parameters could lead to the formation of Al_4_C_3_ [18]. Therefore, in order to ensure the stability of thermal management materials, the preparation method and process parameters must be strictly optimized and controlled.

An LSS technology has been developed based on the principles of powder metallurgy and semi-solid thixoforming [19,20], which has garnered significant attention. Characterized by a low heating temperature and brief holding time, LSS technology prevents the formation of Al_4_C_3_ with deteriorating properties at the interface [21]. Additionally, by leveraging the differential flowability of solid and liquid phases under stress, LSS enables the fabrication of diamond/Al composite shells with a graded distribution of diamond particles and thermophysical properties [22]. This graded distribution addresses the specific needs of different parts of the shell, enhancing the thermal stability of the integrated circuit module.

The TC of diamond/Al composites is primarily influenced by the interface bonding between the Al matrix and diamond particles. Impurities, holes, and other defects at the interface can induce phonon-boundary scattering, reducing the mean free path of phonons and then deteriorating the TC of the composite [4]. Diamond coating represents a cost-effective and efficient modification method, acting as a bridging agent between diamond and Al [23]. Our previous studies have explored the use of various coating layers, such as Cu [19], Ni [20], and Cr [21], to enhance interfacial bonding in LSS-fabricated diamond/Al composites. Recent studies show that Ti coating, a carbide-forming element, is adapted to form an interfacial layer between the matrix and reinforces and strengthens the interfacial bonding [24,25]. However, the influences of Ti coating on the mechanical properties and TC of diamond/Al composite fabricated by the LSS process have not been explored yet. In addition, there are no detailed studies to analyze the effects of composites fabricated with different coatings on bending strength.

This study employed vacuum ion plating to deposit a Ti coating on the surface of diamond particles, utilizing LSS to fabricate diamond/Al composites with a reinforcement phase volume of 40%. The interfacial characterization, corresponding bending property, and TC of the diamond/Al composites have been improved by the Ti coating. The theoretical TC of the diamond/Al composite was calculated using the DEM and Maxwell prediction model.

## 2. Materials and Methods

### 2.1. Materials

For this study, industrial-grade Al powder with a 99.81% purity (by mass) and an average particle size of 37 μm (Zhengzhou Aerospace Aluminum Co., Ltd., Zhengzhou, China) was used. Additionally, MBD-4 grade synthetic single-crystal diamond particles with an average size of 106 μm (Henan Huanghe Whirlwind Co., Ltd., Changge, China) were selected as the primary raw materials. The preferred alternative for diamond/Al composite interface layers is a nanoscale coating layer with high sound velocities, such as Ti, Cr, and other metals [26]. With the increase in coating thickness, the tensile, compressive, and bending strength of diamond/Al composites gradually increase [4], but the TC of the composite increases first and then decreases [27]. On the premise of improving the interface bonding, the coating thickness should be as low as possible to reduce the interface thermal resistance [24]. However, it is difficult to control the layer thickness below 50 nm. The investigation involved the purchase of Ti-coated diamond particles that were manufactured utilizing the vacuum ion plating process. Based on the expected duration of the coating process, the thickness of the Ti coating was approximately 100 nm.

### 2.2. Fabrication of Diamond/Al Composites

**Figure 1:**
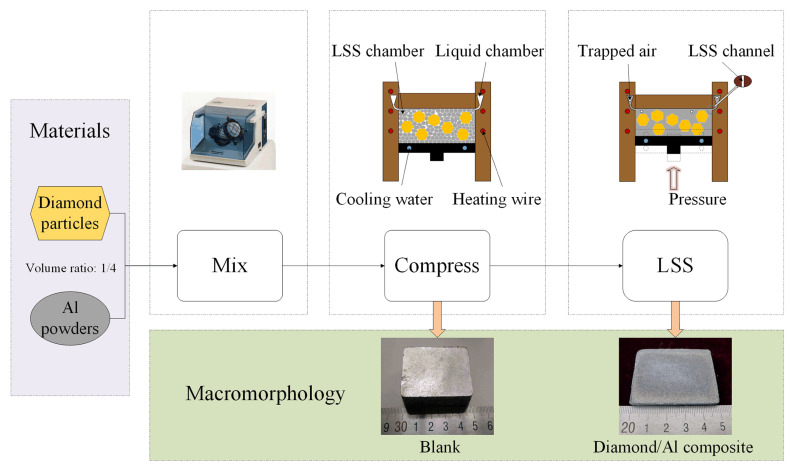
Schematic diagram of the LSS process.

The volumetric fraction of diamond in this composite was 40%. Figure 1 depicts a schematic diagram illustrating the LSS process. The experimental procedure can be articulated as follows: Initially, the diamond particles and Al powder were mixed in a 1:4 volume ratio for 8 h using a Turbula Shaker/Mixer (Model T2C, Glen Mills, PA, USA) to ensure uniform distribution. Furthermore, the homogenously mixed powder was placed into a mold and subjected to compression to form a blank using a cold-pressing technique in a four-column press (Model YQ28-100, Wodda, Zaozhuang, China) at a pressure of 300 MPa for 1 min. The blank measured 6.6 × 38 × 48 mm. Subsequently, the blank was moved to a custom-made LSS mold system and heated until it reached a stage where the liquid and solid components were blended and melted together. The detailed heating process was as follows: the mixture was initially heated at a rate of 20 °C/min to 450 °C and held for 20 min, and then heated for a second time at the same rate to 683 °C, where it was maintained for 40 min. In the fourth step, the molten metal was squeezed into the liquid chamber via the 2 mm LSS channel using a piston under a pressure of 60 MPa, and the diamond particles were completely trapped in the LSS chamber. Ultimately, under the action of cooling water, the slurry in the LSS chamber solidified layer by layer to form a diamond/Al composite with a dimension of 3 × 40 × 50 mm. Throughout the LSS process, hydrogen gas was employed to prevent the oxidation of the Al powder.

### 2.3. Characterization

The diamond/Al composites underwent processing using a laser cutting machine and a diamond wheel grinder. The three-point bending strength of the composites with a dimension of 3 × 4 × 25 mm was examined by an RGM-3010 electronic universal testing machine (Shenzhen, China) at room temperature. The morphologies of the diamond particles, the surface structure, and the three-point bending fractography of the diamond/Al composites were observed by an EVO-18 scanning electron microscope (SEM, Zeiss, Oberkochen, Germany). The distributions of elements across the coating were determined by a JXA-8230 electron microprobe analyzer (EMPA, JEOL, Tokyo, Japan). The phase composition of the composites was certified by an Advance D8 X-ray diffractometer (XRD, Bruker, Saarbruecken, Germany) using Cu Ka radiation at 40 kV and 35 mA. The 2θ scans were executed between 20° and 80° at a scanning speed of 4°/min. In addition, the presence of Al_4_C_3_ was further confirmed by careful scanning between 30° and 45° at a scanning speed of 0.25°/min. Impurity elements such as N, H, and B are often present in diamond particles, with N being the most common. The N content of diamond particles was evaluated by a Nicolet iN10 MX Fourier transform infrared spectrometer (RTIR, ThermoFisher, Waltham, MA, USA). The thermal diffusion (α) of the composites with dimensions of φ 12.7 × 3 mm was measured by an LFA laser flash thermophysical machine (Netzsch, Selb, Germany) at room temperature. The TC value of composites was obtained by an equation: TC = ρ × α × C_p_, where ρ (density) was determined by the Archimedes principle and C_p_ (specific heat capacity) was obtained based on the theoretical calculation. The C_p_ of the composite is equal to the respective C_p_ of the matrix and reinforcement phase multiplied by the corresponding volume fractions.

## 3. Results and Discussion

### 3.1. Microstructure of Diamond/Al Composites

**Figure 2:**
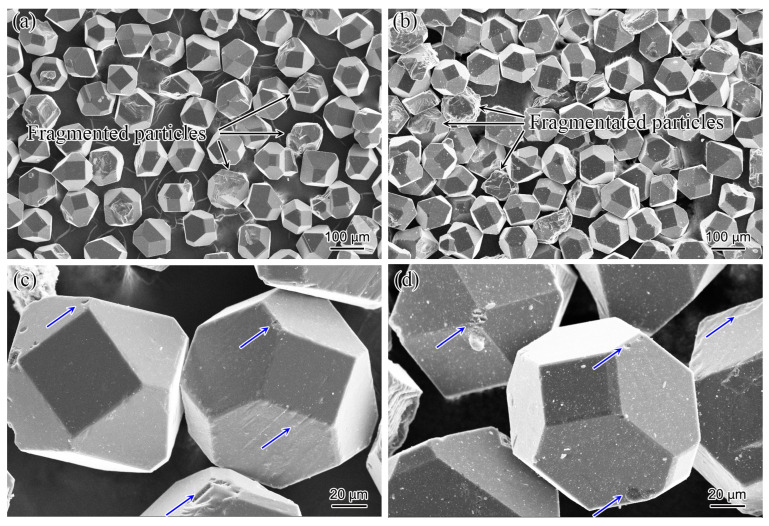
Morphologies of the diamond particles: (**a**) without Ti coating; (**b**) with Ti coating; (**c**) with surface defects; (**d**) with surface defects covered by Ti coating (the defects of diamond surface as shown by blue arrows).

The morphologies of the diamond particles without or with Ti coating are shown in Figure 2. Figure 2a,b illustrates the presence of partially fragmented particles in both types of diamond particles, and Figure 2c,d demonstrates the surface defects (shown by blue arrows) of the diamond particles that have been entirely covered by Ti coating. The metal coating could diffuse into the Al matrix and then form intermetallic compounds with Al, confirmed in diamond/Al with a TiC coating [26]. The Ti coating can improve the interfacial bonding force and enhance the interface conductivity, which is an effective way to fabricate diamond/matrix composites with high TC.

### 3.2. Microstructure of Diamond/Al Composites

**Figure 3:**
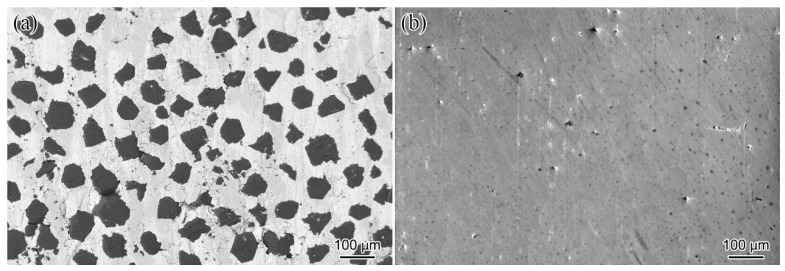
Microstructure of materials fabricated by LSS process: (**a**) diamond/Al composite; (**b**) separated liquid phase.

The microstructure of the diamond/Al composite fabricated by LSS technology is presented in Figure 3a. The synthetic diamond particles without Ti coating were uniformly distributed in the Al matrix, and no spalling of diamond particles was viewed. However, the microstructure of the composite cannot elucidate the behavior of interfacial bonding. Figure 3b displays the microstructure of the separated liquid phase. It can be seen that no diamond particles were discovered in the separated liquid phase, indicating the LSS channel can eliminate the spillover of diamond particles. This also verifies that the LSS technology can precisely fabricate diamond/Al composites with varying volume fractions of reinforcement phase by adjusting the size of the LSS chamber and liquid chamber (as depicted in Figure 1).

**Figure 4:**
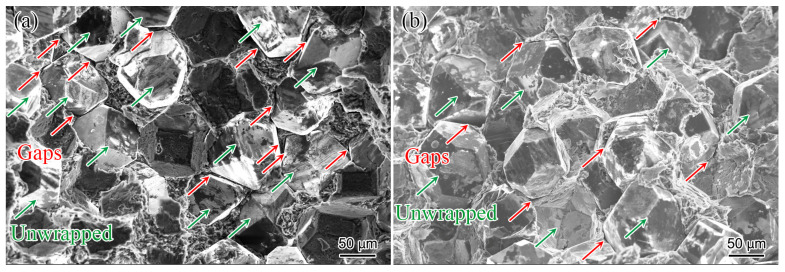
Fractography of the diamond/Al composites: (**a**) without Ti coating; (**b**) with Ti coating.

Fractography can determine whether the bonding state at the interface is of a mechanical or metallurgical nature. Figure 4 shows the fractography of the diamond/Al composites under three-point bending testing, both with and without Ti coating. The plastically deformed Al matrix exhibited network-like dimples on the fracture surface, which is a typical ductile behavior of pure metals. In the area denoted by the green arrows, part of the diamond particles were unwrapped by the Al matrix, indicating a weak interfacial bonding strength between the diamond and Al. However, in the area specified by the red arrows, the Al of the composite was not fully covered with the diamond particles, resulting in the formation of a gap. The gap, i.e., the arch bridge phenomenon, hinders the transmission of phonons at the interface between the matrix and reinforcement, reducing the TC of the composite [4,28]. By comparing Figure 4a,b, it can be seen that the Ti coating on the diamond surface can effectively reduce the phenomenon of unwrapping and gaps, increasing the interface bonding strength of the diamond/Al composite, which also improves the relative density of the composite. Ti coating can improve the interface bonding state of diamond/Al composites, which is consistent with the results of fractography of composites prepared by an alternative method [25]. High relative density is a prerequisite for obtaining diamond/Al composite materials with high TC [13,16].

### 3.3. Interfacial Characteristics of Ti-Diamond/Al Composites

**Figure 5:**
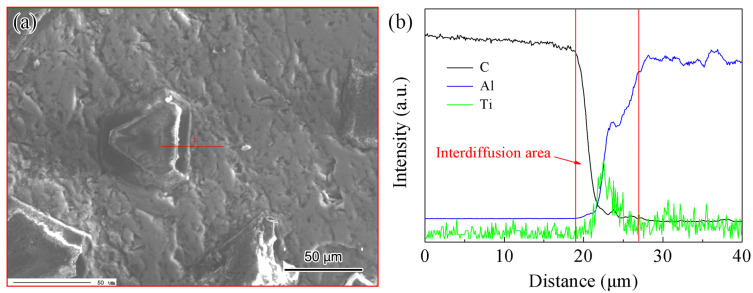
Interface element distribution of the diamond/Al composite with Ti-coated diamond particles: (**a**) SEM image; (**b**) EMPA map.

Figure 5 shows the distribution of interfacial elements in the diamond/Al composite with Ti-coated diamond particles using EMPA. Due to the limited solid solubility of diamond and Al to dissolve into each other, an interdiffusion area of approximately 8 μm was generated, as shown in the red line area in Figure 5b. The Ti coating on the surface of diamond particles diffused toward the Al side, demonstrating that metallurgical bonding has been formed in the interface region between diamond and Al, which enhances the performance of the composites [21,23].

**Figure 6:**
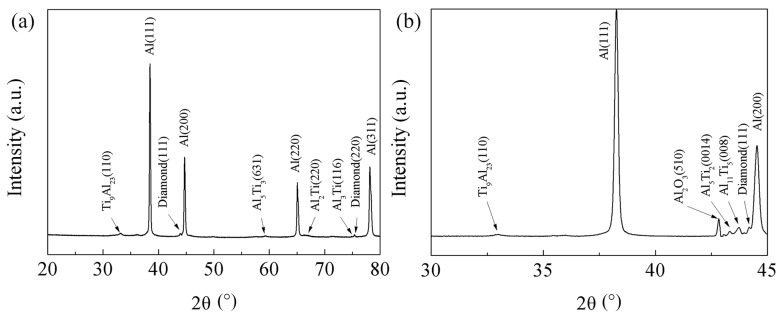
XRD patterns of the diamond/Al composite with Ti-coated diamond particles in different diffraction angle ranges: (**a**) between 20° and 80°; (**b**) between 30° and 45°.

Figure 6 manifests the XRD patterns of the diamond/Al composite with Ti-coated diamond particles. The patterns were obtained in different diffraction angle ranges at a scanning speed of 4 and 0.25°/min. Figure 6a demonstrates the formation of Al_3_Ti, Al_2_Ti, Al_5_Ti_3_, and Ti_9_Al_23_ intermetallic compounds in the diamond/Al composite with Ti-coated diamond particles, which results from the long-term reaction between Ti coating and Al matrix at the fabrication temperature during the LSS process. The intermetallic compounds tightly connect the Al matrix with diamond particles, improving the bonding strength between the Al and diamond phases [21,25]. Figure 6b indicates that the Al_4_C_3_ phase was not detected even at the scanning speed of 0.25°/min. Additionally, a trace peak of Al_2_O_3_ was discovered, which can be attributed to the oxidation of the composite during the storage. A continuous and well-bonded interfacial structure without the Al_4_C_3_ phase is the key to improving the TC and stability of diamond/Al composites [18]. The EMPA and XRD confirm that Ti coating on the diamond surface is conducive to forming a well-bonded interface.

### 3.4. Bending Strength of Diamond/Al Composites

**Figure 7:**
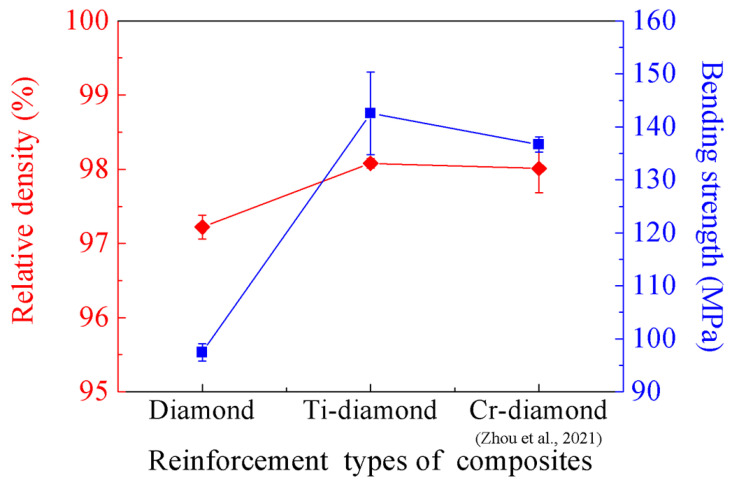
Variation in the relative density and bending strength of diamond/Al composites fabricated by the LSS process [21].

Figure 7 compares the relative density and bending strength of the diamond/Al composites fabricated by the LSS process in this study and those reported in the literature [21]. Compared with uncoated diamond/Al composites, the relative density of Ti-diamond/Al composites increased from 97.22% to 98.08% with a growth rate of 0.88%, and the bending strength increased from 97.46 MPa to 142.54 MPa with a growth rate of 46.25%. Meanwhile, the bending fractography in Figure 4 shows that a more interfacial gap is observed between the uncoated diamond and the Al matrix. The separation occurs due to the significant disparity in the CTE between diamond (1.0 × 10^−6^/K) and Al (23.0 × 10^−6^/K) when undergoing the cooling process. Therefore, the low relative density of uncoated diamond/Al composites is attributed to the abundance of gaps surrounding the diamond particles. In other words, the Ti coating plays a vital role in promoting interfacial bonding and improving mechanical properties. Furthermore, certain studies have demonstrated that a thick Ti coating can effectively establish a strong bond between the diamond particles and the Al matrix, hence enhancing the composite interface [25]. By comparing Figure 7, it can also be seen that in the diamond/Al composites prepared by the LSS technology, the bending strength and relative density of the Ti coating on the diamond surface are better than those of the Cr coating. The CTE of Ti is 10.8 × 10^−6^/K, whereas that of Cr is 6.2 × 10^−6^/K. Compared with the Cr coating, the Ti coating, as an intermediate layer, can better buffer the difference in CTE between the diamond and Al matrix, further reducing gaps and increasing the relative density of the diamond/Al composite. Therefore, Ti-coated diamond/Al composites have superior bending strength compared to Cr-coated diamond/Al composites.

### 3.5. Thermal Conductivity of Diamond/Al Composites

**Figure 8:**
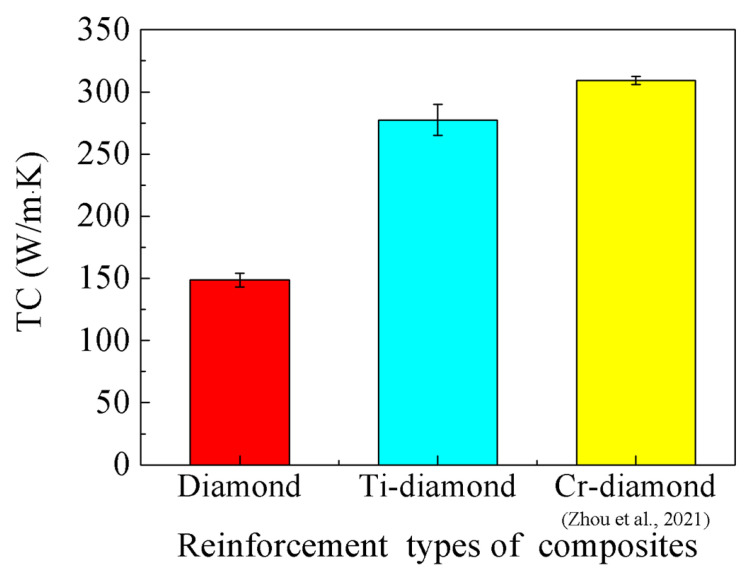
TC of the diamond/Al composite fabricated by the LSS process [21].

Figure 8 illustrates the TC of the diamond/Al composite both without and with Ti coating. The lack of wetting capacity between the diamond and the Al matrix results in a TC of only 149 W/m·K in uncoated diamond particle-reinforced Al matrix composites. In contrast, the TC of Ti-coated diamond/Al composites exhibited a significant increase of 85.9%, reaching a value of 277 W/m·K. Ti coating can significantly improve the interfacial bonding between diamond and Al, leading to an increase in the TC of composites, which is consistent with other study results [24]. It is worth noting that the TC of the Ti-diamond/Al composite is lower than that of the Cr-diamond/Al composite prepared by the same method [21]. This difference in TC could be the reason why the interfacial products of the Ti-diamond/Al composite are more intricate, as evidenced by the detection of intermetallic compounds using X-ray diffraction (XRD) in Figure 6. The complex intermetallic compounds incorporated extra interface layers that act as thermal boundary barriers. So, the interfacial thermal conductance (ITC) and TC of Ti-coated diamond/Al composites decrease significantly with the increase in the thickness of the intermetallic layer, which is due to the very low TC of the intermetallic layer generated by Ti and Al [26]. The thickness of the intermetallic layer reached 8 μm in this study, as shown in the red line area in Figure 5b. In addition, Cr coating has a greater sound velocity compared to Ti coating, which is also the reason for the higher TC of its composite [26]. The interfacial bond between diamond and Al can be improved by the appropriate thickness of the intermetallic layer, which enhances the TC of the composite. However, an excessively thick intermetallic layer with low TC increases the interfacial thermal resistance and thus reduces the TC of the composite [29]. Therefore, it is imperative to meticulously regulate the thickness of the intermetallic layer.

### 3.6. Theoretical Thermal Conductivity of Diamond/Al Composites

**Figure 9:**
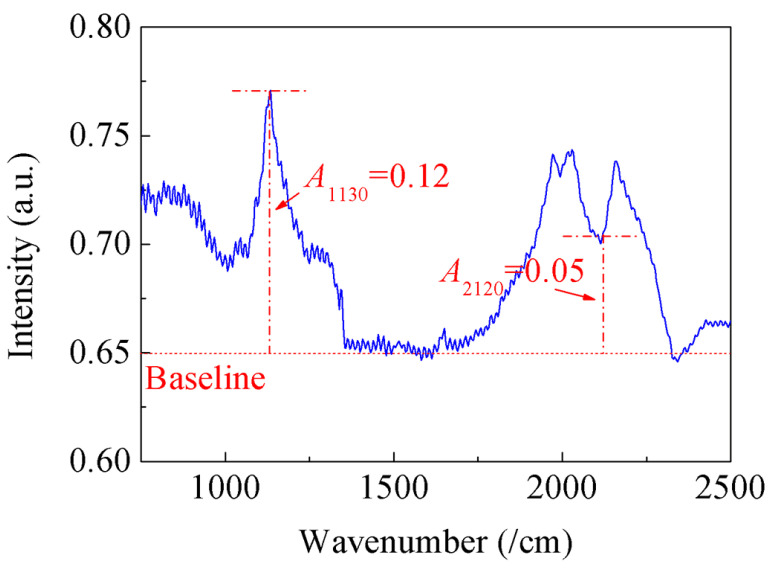
FTIR diffuse reflectance spectrum of the diamond particle.

The heat transfer between metal and non-metal phases depends on the coupling effect of electrons (on the metal side) and phonons (on the non-metal side) [30]. The presence of impurities, holes, and other defects at the interface between the two phases would cause phonon scattering, leading to a reduction in the mean free path of phonons and thus deteriorating the TC of composites [4]. The DEM (differential effective medium) [31] and Maxwell [32] are widely used in the TC prediction for the diamond/Al composites by comprehensively considering many factors, such as ITC and acoustic mismatch theory [33]. These models can be expressed as

(1) $${\left(\frac{\mathrm{TC}}{{\mathrm{TC}}_{m}}\right)}^{\frac{1}{3}}\left(1\;-V_{d}\right)=\frac{\frac{{\mathrm{TC}}_{d}^{\mathrm{eff}}}{{\mathrm{TC}}_{m}}\;\;-\frac{\mathrm{TC}}{{\mathrm{TC}}_{m}}}{\frac{{\mathrm{TC}}_{d}^{\mathrm{eff}}}{{\mathrm{TC}}_{m}}-1},$$

(2) $$\mathrm{TC}=\frac{{\mathrm{TC}}_{m}\;\times \;[2\;\times \;{\mathrm{TC}}_{m}+{\mathrm{TC}}_{d}^{\mathrm{eff}}+2\;\times \;({\mathrm{TC}}_{d}^{\mathrm{eff}}-{\mathrm{TC}}_{m})\;\times \;V_{d}]}{2\;\times {\mathrm{TC}}_{m}+{\mathrm{TC}}_{d}^{\mathrm{eff}}-\;({\mathrm{TC}}_{d}^{\mathrm{eff}}-{\mathrm{TC}}_{m})\;\times \;V_{d}},$$

where ${\mathrm{TC}}_{m}$ and ${\mathrm{TC}}_{d}^{\mathrm{eff}}$ are the TC of the Al and the effective TC of the diamond, respectively, and $V_{d}$ is the volume fraction of diamond particles. Considering the influence of ITC and diamond particle size on the TC of reinforcement, ${\mathrm{TC}}_{d}^{\mathrm{eff}}$ can be calculated as

(3) $${\mathrm{TC}}_{d}^{\mathrm{eff}}=\frac{{\mathrm{TC}}_{d}^{\mathrm{in}}}{1+\frac{{\mathrm{TC}}_{d}^{\mathrm{in}}}{r\;\times \;\mathrm{ITC}}},$$

where *r* and ${\mathrm{TC}}_{d}^{\mathrm{in}}$ are the average radius and TC of single-crystal diamond particles, respectively. The ${\mathrm{TC}}_{d}^{\mathrm{in}}$ of the synthetic single-crystal diamond has a linear relationship with its N element content and decreases with the increase in N content ([*N*]) [34,35]. According to the equation (i.e., [*N*] = 5.5 × 25 × $A_{1130}/A_{2120}$), the N content can be estimated from the intensity ratio of the relative absorption coefficient (*A*) [36]. Figure 9 shows the intensity of the absorption peak at wavenumbers of 1130 and 2120/cm. The N content of diamond particles used in this experiment was about 330 ppm, and the TC of diamond was about 1121 W/m·K, according to the equation (${\mathrm{TC}}_{d}^{\mathrm{in}}=2200-3.27[N]$) [34].

Then, the ITC between the Al matrix and diamond particles can be described as

(4) $$\mathrm{ITC}=\frac{1}{\;4}\;\times \;\rho_{m}\;\times \;c_{pm}\;\times \;C_{\mathrm{Dm}}\;\times \;\eta_{1-2},$$

where $\rho_{m}$, $c_{pm}$, and $C_{\mathrm{Dm}}$ are the mass density, specific heat capacity, and sound velocity for the matrix. Then, $\eta_{1-2}$ is the average transmission coefficient of phonons across the interface from Al to diamond, which can be denoted as

(5) $$\eta_{1-2}\;=\frac{2\;\times \;Z_{m}\;\times \;Z_{d}}{{\left(Z_{m}\;+Z_{d}\right)}^{2}}\times \;(\frac{C_{\mathrm{Dm}}}{C_{\mathrm{Dd}}}),$$

where $Z_{m}$ and $Z_{d}$ are the phonon impedance (according to the equation: *Z* = $\rho C_{D}$) for the matrix and reinforcement, respectively, and $C_{\mathrm{Dd}}$ is the sound velocity for the reinforcement. The sound velocity of the matrix and the reinforcement can be calculated according to the Debye sound velocity ($C_{D}$), and the theoretical calculation can be established as

(6) $$C_{D}=\frac{1}{\sqrt{\frac{1}{2}\;\times \;(\frac{1}{C_{l}^{2}}+\frac{1}{C_{t}^{2}})}},$$

where $C_{l}$ and $C_{t}$ are longitudinal phonon velocity and transversal phonon velocity, respectively. Table 1 represents the physical parameters of raw materials for calculation in this study. Inputting the data into Equations (1) and (2) calculates the theoretical TC of DEM and Maxwell as 389 and 382 W/m·K, respectively, and the measured values reach 71.2 and 72.5% of the theoretical values, respectively. The effect of coating on interfacial bonding leads to the improvement of the TC of the composite. However, the TC of the composite is still far from the theoretical models of DEM and Maxwell due to the intrinsic interfacial thermal resistance of the coating.

Possible factors contributing to the substantial discrepancy between the actual measured values and the calculated values of thermal conductivity are as follows: (i) The theoretical model is based on the premise that diamond particles are spherical, while in reality, high-quality diamond particles are hexoctahedrons, and the shape factor will reduce the interfacial heat transfer coefficient. (ii) The effect of Ti coating on diffusion rate is neglected in the calculation of ITC. (iii) The intermetallic compounds formed at the interface with low intrinsic TC deteriorate the interfacial thermal resistance of the composite. (iv) The theoretical model does not consider the effect of internal defects on the TC of the composite, and internal defects are the inevitable structure of the composite.

## 4. Future Research Directions

The design of the thermal conduction path at the interface between matrix and reinforcement will directly affect the TC property of the diamond/Al composites. The metal matrix alloying method has been proven to be able to utilize the diffusion dynamics of matrix alloy elements to build parallel structural carbides, reducing the interface thermal resistance [39]. However, the alloying elements in the matrix act as additional interface thermal resistance, which offsets the effect of part of the parallel structure in reducing the total thermal resistance. Diamond coating is the only second interface modification method in this field. How to synthesize discontinuous metal coating in situ on the diamond surface and then prepare composites with parallel structure thermal conduction paths is the future development direction.

## 5. Conclusions

The independently developed liquid–solid separation (LSS) technology has been applied to prepare 40 vol.% diamond particle-reinforced diamond/Al composites. The interfacial characteristics, bending strength, and thermal conductivity (TC) of the diamond/Al composites without and with a thickness of 100 nm of Ti coating were evaluated comprehensively. The main conclusions can be summarized as follows:

(1) The LSS technology, characterized by its low heating temperature and short holding time, prevents the formation of Al_4_C_3_ at the interface of diamond/Al composites, hence preserving their mechanical and thermophysical properties.
(2) The inclusion of Ti coating formed Al_3_Ti, Al_2_Ti, Al_5_Ti_3_, and Ti_9_Al_23_ intermetallic compounds, resulting in metallurgical bonding between diamond and Al, improving the interfacial bonding strength and TC of the diamond/Al composites.
(3) The TC increased from 149 W/m·K for the diamond/Al composite to 277 W/m·K for the diamond/Al composite with Ti-coated diamond particles, with a growth rate of 85.9%.
(4) A comparison of the present Ti coating composite with previously reported Cr coating composite was performed on the features. The application of the Ti coating can increase the bending strength of the composite, while the Cr coating can enhance the TC of the composite.
(5) The Ti coating promotes interfacial bonding but also introduces extra interfacial thermal resistance. Combined with the idealization of the model design, results for TC reached only 71.2% with the DEM model and 72.5% with the Maxwell model, respectively.

## Figures and Tables

**Table 1:** Physical parameters of raw materials [32,37,38].

Material	Phonon Velocity		*C*_D_ (m/s)	*η*_l−2_	ITC (W/m^2^·K)	$c_{p}$ (J/g·K)
	*C*_l_ (m/s)	*C*_t_ (m/s)				
Al	6240	3040	3865	0.019	4.43 × 10^7^	0.895
Diamond	20,000	12,300	14,817			0.500

## Data Availability

The datasets used and analyzed during the current study are available from the corresponding author upon reasonable request.
